# Protein Representation in Metric Spaces for Protein Druggability Prediction: A Case Study on Aspirin

**DOI:** 10.3390/ph18111711

**Published:** 2025-11-11

**Authors:** Jiayang Xu, Shuaida He, Yangzhou Chen, Xin Chen

**Affiliations:** 1 Department of Statistics and Data Science, Southern University of Science and Technology, Shenzhen 518055, China; 12313323@mail.sustech.edu.cn (J.X.); 12331110@mail.sustech.edu.cn (Y.C.); 2 Department of Statistics and Actuarial Science, The University of Hong Kong, Hong Kong, China; sdhe@hku.hk

**Keywords:** protein druggability, biological interpretability, non-euclidean data, KNN classifier, protein representation

## Abstract

**Background:** Accurately predicting protein druggability is crucial for successful drug development, as it significantly reduces the time and resources required to identify viable drug targets. However, existing methods often face trade-offs between accuracy, efficiency, and interpretability. This study aims to introduce a lightweight framework designed to address these challenges effectively. **Methods:** We present a lightweight framework that embeds proteins into four biologically informed, non-Euclidean metric spaces, derived from analyses of amino acid sequences, predicted secondary structures, and curated post-translational modification (PTM) annotations. These representations capture key features such as hydrophobicity profiles, PTM densities, spatial patterns, and secondary structure composition, providing interpretable proxies for structure-related determinants of druggability. This approach enhances our understanding of protein functionality while improving druggability predictability in a biologically relevant context. **Results:** Evaluated on an Aspirin-binding protein dataset using leave-one-out cross-validation (LOOCV), our distance-based ensemble achieves 92.25% accuracy (AUC = 0.9358) in the whole-protein setting. This performance significantly outperforms common sequence-only baselines in the literature while remaining computationally efficient. **Conclusions:** On a refined single-chain subset, our framework demonstrates performance comparable to established feature engineering pipelines, highlighting its potential effectiveness in practical applications. Together, these results strongly suggest that biologically grounded, non-Euclidean embeddings provide an effective and interpretable alternative to resource-intensive 3D pipelines for target assessment in drug discovery. This approach not only enhances our ability to assess protein druggability but also streamlines the overall process of target identification and validation.

## 1. Introduction

Druggability means the probability that a target can be modulated by small molecules [1,2,3,4]. For many structural and computational biologists, a longstanding central question has been predicting a protein’s capacity to bind small molecules or drugs effectively [5].

The integration of deep learning (DL) models has resulted in significant progress in the druggability prediction field [6,7]. Among these methods, AlphaFold has emerged as a transformative tool, delivering unprecedented accuracy in protein structure prediction [7]. Unfortunately, AlphaFold still has constraints in druggability prediction because not all relevant physicochemical and biological factors are fully considered, necessitating further verification and correction [8]. Another DL-based methodology, given by [9], applies several neural networks, including Convolutional Neural Network (CNN) models and Residual Neural Network (ResNet) models, showing reasonable progress in model performance but lacking interpretability for drug designers.

**Figure 1:**
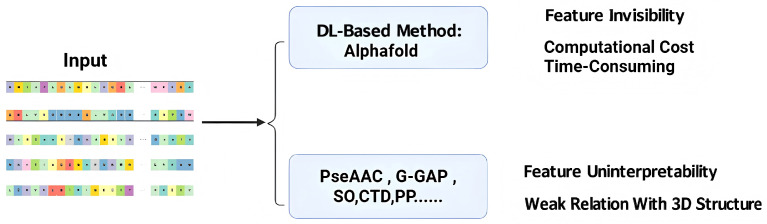
Current methods’ workflow and their disadvantages.

Another line of research, based on traditional machine learning (ML) models, focuses solely on feature engineering on amino acid sequences [10,11]. For instance, amino acid composition (AAC) [12,13] quantifies the frequency of each amino acid, but it fails to adequately link these frequencies to the three-dimensional structure of proteins, significantly limiting its utility in structural analysis. Likewise, pseudo-amino-acid composition (PseAAC) [14,15] incorporates some physicochemical properties; however, it also relies solely on the amino acid sequence, rendering it inadequate for providing comprehensive structural insights. The G-GAP method [16] adopts a different approach but lacks sophistication, treating the amino acid sequence as a string of tokens and counting G-GAP dipeptides in a rudimentary, natural language processing (NLP)-like fashion, thus missing the complex nuances of protein structure. Similarly, methods like sequence order (SO) [17], composition–transition–distribution (CTD) [15], physicochemical property (PP) [18], and Hidden Markov Model (HMM) [19] reflect the narrow focus solely on amino acid sequences. All these approaches underscore a crucial issue—the absence of structure-based feature design. Relying exclusively on amino acid sequence features neglects biologically relevant criteria for screening druggable proteins, ultimately impeding accurate predictions in druggability assessments. Further details are provided in Figure 1.

Given the current challenges in early-stage drug discovery, where a lack of structure-based features hinders the effective characterization of potential protein targets, we present a novel approach to protein representation that directly addresses this critical limitation. Our methodology leverages the essential biological information embedded in amino acid sequences, the locations of post-translational modification (PTM) sites, and secondary structure proportion, utilizing appropriate non-Euclidean metrics to evaluate the distance between proteins. This approach enables a more comprehensive and biologically functional representation of proteins, which is crucial for assessing protein druggability. When benchmarked on the critical task of predicting protein druggability, our feature-design framework demonstrates substantial improvements in predictive performance and robustness over existing methods. This result not only validates the strong correlation between our designed features and a protein’s structural and functional attributes but, more importantly, opens up new, more efficient avenues for identifying promising drug targets, directly impacting the fields of computational drug discovery and therapeutic protein engineering.

## 2. Results

### 2.1. Case Study: Aspirin

**Figure 2:**
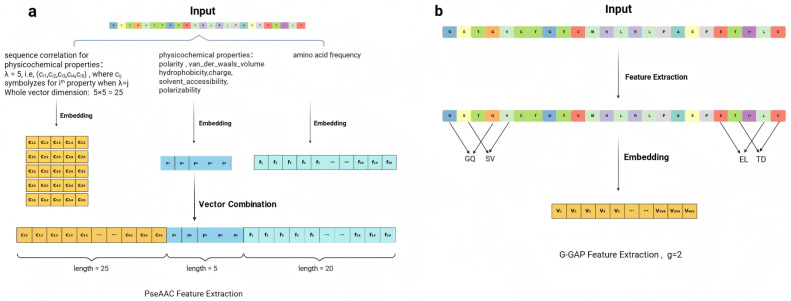
Schematic of feature extraction workflows for two comparative methods. (**a**) A variant of pseudo-amino acid composition (PseAAC). Three components are extracted and concatenated: (1) a 25-dimensional vector of sequence-order correlation factors derived from 5 physicochemical properties at 5 tiers (λ=5); (2) a 5-dimensional vector of physicochemical properties; (3) a 20-dimensional vector of amino acid frequencies. (**b**) Gapped-dipeptide frequency (g=2). Dipeptide patterns with a gap of 2 amino acids are extracted and their frequencies counted, yielding a 400-dimensional feature vector (20×20 possible combinations).

We apply our methodology to the Aspirin dataset to demonstrate its effectiveness. Our evaluation focuses on both single-chain and complete protein datasets, encompassing the entire dataset for a robust analysis. Notably, we outline our sample disassembly and chain-wise labeling strategy, a common approach for disassembling and analyzing a multi-chain protein complex. Our methods show a superior performance compared with other conventional feature engineering methods with several ML models, including Random Forest (RF), K-nearest neighbors (KNN), Logistic Regression, and Support Vector Machine (SVM), as shown in Figure 2.

#### 2.1.1. Data Disassembly via Unsupervised Clustering

In the context of protein disassembly, we first deconstruct multi-chain protein complexes into their constituent individual chains. These chains are then subjected to an unsupervised K-means clustering algorithm, using the $l_{1}$ norm of their secondary structure proportions as the sole feature. We chose this strategy because secondary structure often indicates structural and functional relatedness more reliably than sequence alone. This approach allows us to group chains based on functional potential, increasing the effective sample size and capturing the relevant diversity of potential drug–target binding sites.

**Figure 3:**
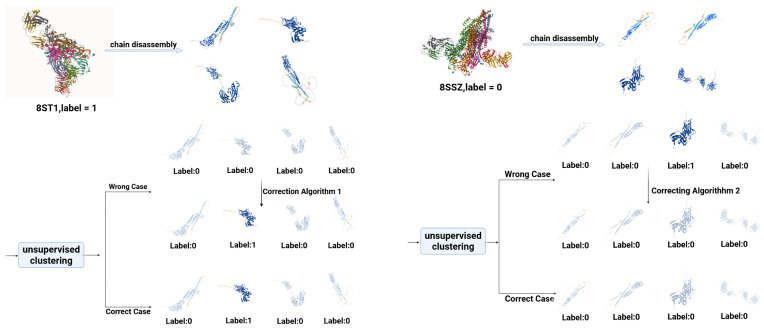
Principle of protein complex disassembly and Correcting Algorithms. Multi-chain complexes are disassembled into individual chains and subjected to unsupervised clustering. **Left** (**Correcting Algorithm 1**): For a known drug-binding complex (e.g., 8ST1, label = 1), at least one chain must be labeled positive. If clustering fails to assign any positive label, the chain with the highest secondary structure similarity to the positive centroid is corrected to positive. **Right** (**Correcting Algorithm 2**): For complexes with no drug annotations (e.g., 8SSZ, label = 0), all chains are enforced as negative. Any positive labels assigned by clustering are reverted to negative.

While this initial clustering provides a strong, data-driven foundation, we further enhance the biological relevance and logical consistency of the labels through a rule-based refinement process. This process involves two “Correcting Algorithms,” which integrate established biological ground truths into the unsupervised learning output, as illustrated in Figure 3. The details are as follows:

Correcting Algorithm 1: For any multi-chain protein complex annotated as positive (e.g., an Aspirin-binding complex), we enforce the logical constraint that at least one of its constituent chains must carry a positive label. If the initial clustering does not reflect this, the algorithm identifies the chain with the highest secondary structure similarity (measured by the smallest $l_{1}$ distance) to the positive cluster centroid and aligns its label to positive. This step ensures that known positive biological information is faithfully represented in our dataset.

Correcting Algorithm 2: For any multi-chain protein complex with no known druggability annotations, we apply the constraint that all its chains are confirmed as negative. If the initial clustering assigns a positive label to any such chain, this algorithm reverts the label. This refinement step maintains the purity of our positive set by preventing the inclusion of labels from uncharacterized complexes.

The value of this two-step labeling strategy—combining unsupervised clustering with rule-based refinement—is empirically validated through a rigorous ablation study. We compared our Full Model (K-means + Correcting Algorithm) against an Ablated Model (using only raw K-means labels without corrections). As shown in Table 1, the correcting algorithm provides a consistent but modest performance improvement across all metrics. Our Full Model achieved an accuracy of 92.25%, whereas the Ablated Model retained a competitive accuracy of 89.44%.

Critically, this 2.81% absolute accuracy difference demonstrates that our correcting algorithms do not introduce significant positive bias. The Ablated Model’s robust performance (89.44% accuracy, 89.23% precision, 81.92% recall, and 84.70% F1 score) confirms that the core predictive power resides in our biologically informed metric space representations rather than in label manipulation. The correcting algorithms serve as a refinement step that addresses edge cases where K-means clustering mislabels chains in multi-chain complexes—particularly when druggable chains co-cluster with non-druggable partners due to sequence or structural similarity.

The minimal performance degradation when corrections are removed (retaining 96.95% of the Full Model’s accuracy) provides strong empirical evidence that our method does not artificially inflate results. Instead, the 2.81% improvement reflects a meaningful resolution of labeling ambiguities inherent to unsupervised clustering, while the model’s generalization capability remains fundamentally driven by the quality of the metric space features.

**Figure 4:**
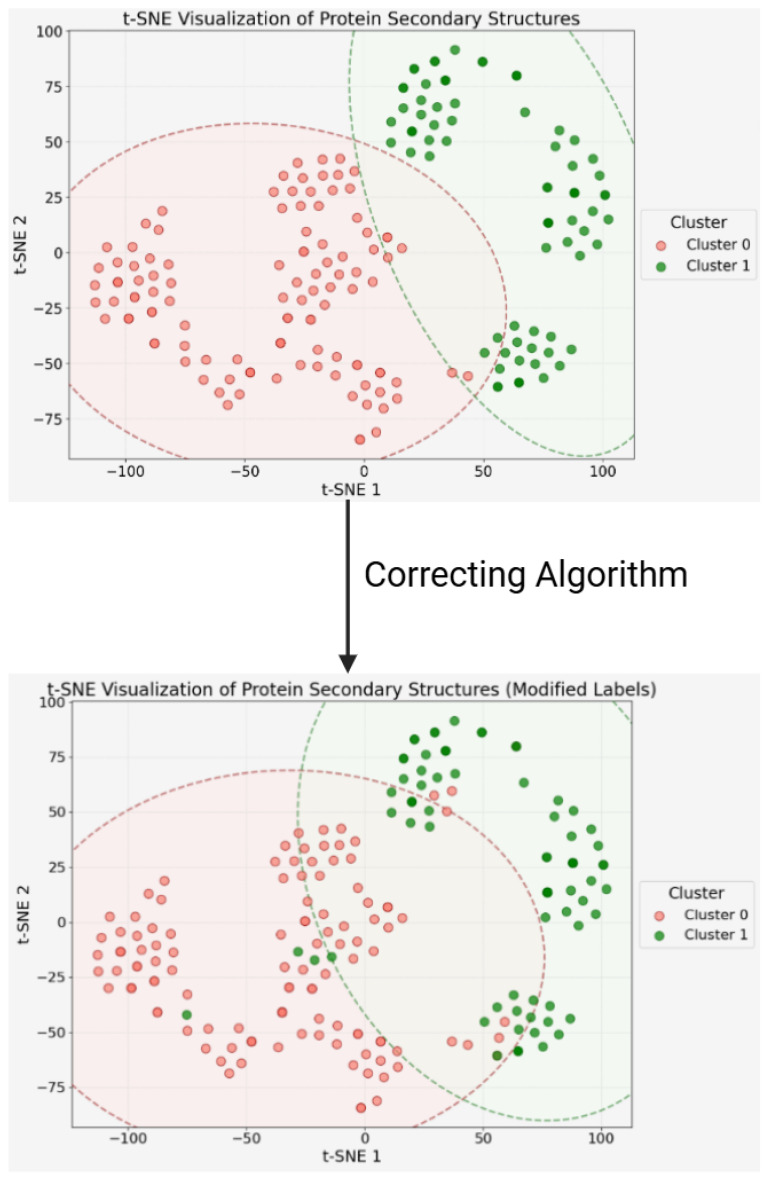
The result after applying chain-disassembling, unsupervised clustering, and correcting algorithms.

After disassembling the protein complexes and labeling the single chains, we get the result of sample distribution, as given in Figure 4.

Furthermore, the principle of this correction strategy serves a crucial downstream purpose that goes beyond classification accuracy. A primary goal of our work is to enable the “retroactive localization” of promising targets within their original multi-chain complexes. By ensuring that chains originating from known positive complexes are correctly labeled as “positive,” our algorithms guarantee that these vital complexes are not lost during analysis. This allows researchers to reliably trace a high-scoring single chain back to its biologically relevant, multi-subunit origin for further investigation. The “Ablated Model,” despite its reasonable accuracy, would frequently fail at this critical task, rendering it far less useful as a practical discovery tool.

The principle of the methodology employs a “Divide and Conquer” strategy for handling multi-chain protein complexes, focusing on the fact that a positively labeled chain can be traced back and relocated to its corresponding protein complex, indicating that the protein complex displays druggability.

#### 2.1.2. Performance on Single-Chain Protein Dataset

To verify the scientific soundness, effectiveness, and persuasiveness of our method, we initially applied the framework to single-chain protein classification, where the labeling process is unambiguous. This clear context provides a solid foundation for our initial model evaluation.

**Figure 5:**
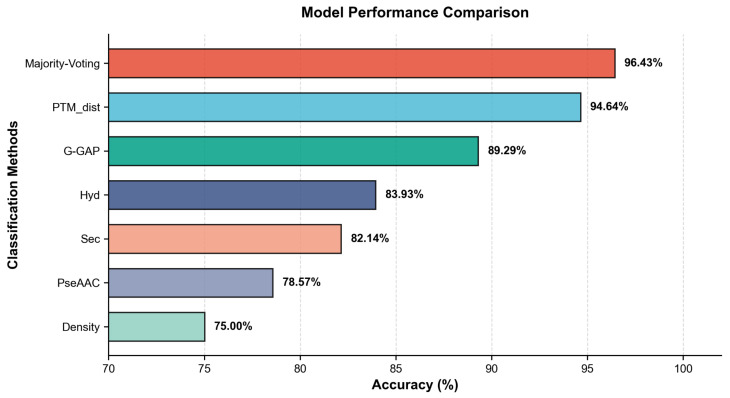
Majority Voting model, basic models, and benchmark models comparison based on KNN classifier.

According to Figure 5, our ensemble model, Majority Voting, delivers the highest accuracy (96.43%), indicating that aggregating heterogeneous predictors effectively exploits complementary error patterns and yields robust generalization. The comparative baselines, G-GAP (89.29%) and PseAAC (78.57%), trail substantially: G-GAP captures some residue-order correlations but lacks sufficient contextual breadth, while PseAAC’s coarse compositional encoding proves least informative. The pronounced margin over both baselines underscores the advantage of our ensemble integration strategy.

**Figure 6:**
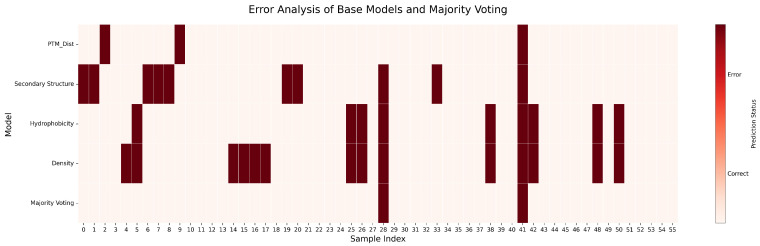
Error analysis of base models and Majority Voting on the single-chain Aspirin protein dataset. Each row represents a model, and each column corresponds to a test sample. Dark red indicates errors; light colors indicate correct predictions.

To dissect the ensemble’s performance gains, we visualized sample-wise prediction errors across all models, as shown in Figure 6. The resulting heat map reveals partially non-overlapping error profiles among the base learners, a key requirement for effective ensemble integration. Notably, the Density model, despite its lower aggregate accuracy, correctly classifies several samples that are consistently misclassified by the other more complex feature sets. This complementary success demonstrates that the Density feature provides unique, non-redundant predictive information. Its inclusion is therefore justified, as it allows the Majority Voting ensemble to correct for correlated errors among the other base models, enhancing overall robustness and accuracy.

**Figure 7:**
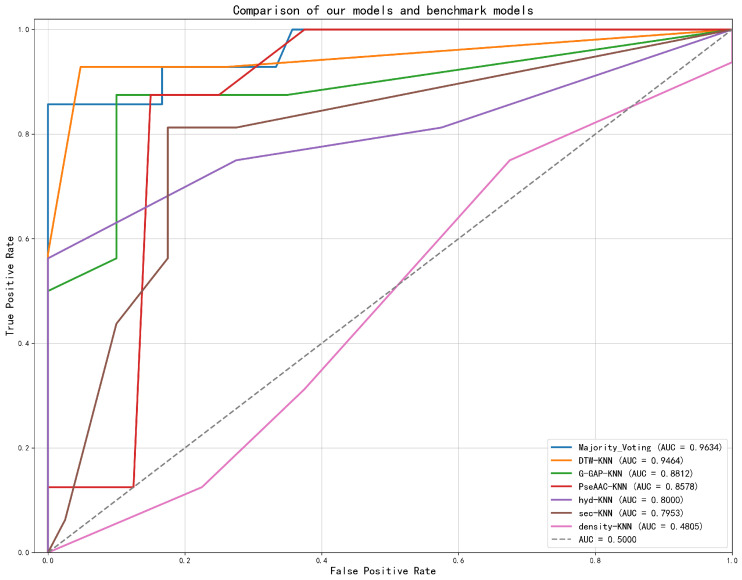
ROC curves comparing the performance of individual feature models (DTW-KNN, G-GAP-KNN, PseAAC-KNN, hyd-KNN, sec-KNN, Density-KNN), the Majority Voting ensemble model, and a random baseline. The Majority Voting model achieves the highest AUC of 0.9634 on the single-chain Aspirin protein dataset.

Receiver Operating Characteristic (ROC) analysis revealed that the proposed Majority Voting ensemble (AUC = 0.9634) substantially outperformed its constituent base models and established benchmarks, such as G-GAP + KNN (AUC = 0.8812) and PseAAC + KNN (AUC = 0.8578), as shown in Figure 7. This highlights the synergistic benefit of ensembling heterogeneous feature sets.

To gain deeper insights into the classification behavior of different feature representations, we conducted a comprehensive analysis of their distance distributions and neighbor relationships on the single-chain protein dataset.

**Figure 8:**
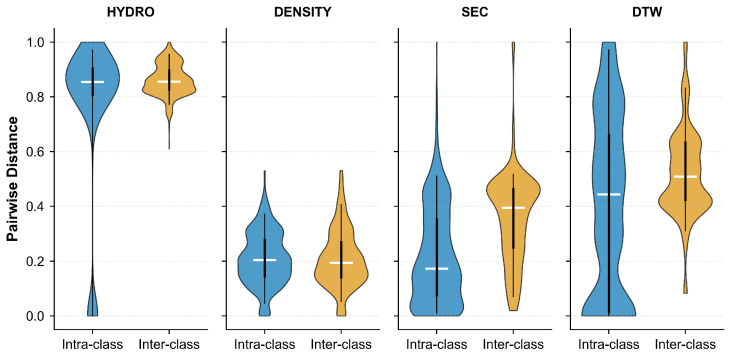
Pairwise distance distributions for different features on the single-chain protein dataset. Each panel compares intra-class (blue) and inter-class (yellow) distances for HYDRO, DENSITY, SEC, and DTW features. Horizontal lines indicate medians.

Figure 8 presents the pairwise distance distributions for four different features: Hydrophobicity (HYDRO), Density (DENSITY), Secondary Structure (SEC), and DTW-based distance (DTW). The violin plots compare intra-class distances (blue) against inter-class distances (yellow) for each feature. Ideally, effective features should exhibit small intra-class distances and large inter-class distances to ensure good separability. As shown in the figure, HYDRO and SEC demonstrate relatively well-separated distributions, with inter-class distances substantially larger than intra-class distances. In contrast, DENSITY and DTW show considerable overlap between the two distributions, indicating limited discriminative power for these features.

**Figure 9:**
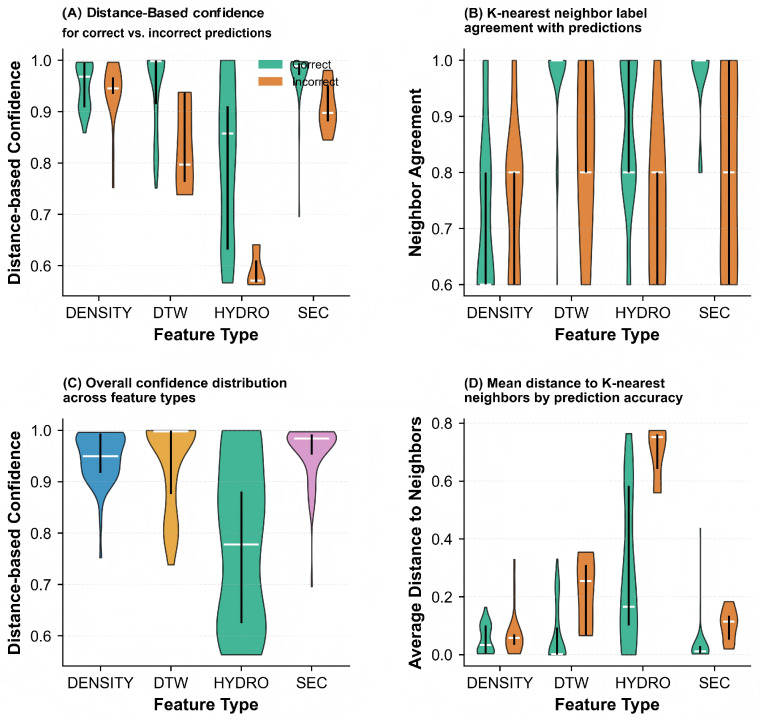
Analysis of classification confidence and neighbor relationships for different features on the single-chain protein dataset. (**A**) Distance-based confidence: Correct (green) vs. incorrect (orange). (**B**) Neighbor agreement distribution. (**C**) Feature-wise confidence comparison. (**D**) Average neighbor distance. Features include DENSITY, DTW, HYDRO, and SEC.

To further investigate the reliability of individual base classifiers, we analyzed the distance-based confidence and neighbor agreement patterns in Figure 9. Panel (A) shows the distance-based confidence distribution, where correct predictions (green) generally exhibit higher confidence scores compared to incorrect predictions (orange) across all features. Panel (B) illustrates the neighbor agreement distribution, which measures the consistency of class labels among nearest neighbors. Higher neighbor agreement typically correlates with correct predictions, particularly for DTW and SEC features. Panel (C) provides a feature-wise comparison of prediction confidence, revealing that DENSITY and DTW maintain consistently high confidence levels, while HYDRO shows greater variability. Panel (D) displays the average distance to neighbors, where correct predictions tend to have smaller average distances, indicating that correctly classified samples are situated in more compact regions of the feature space.

**Figure 10:**
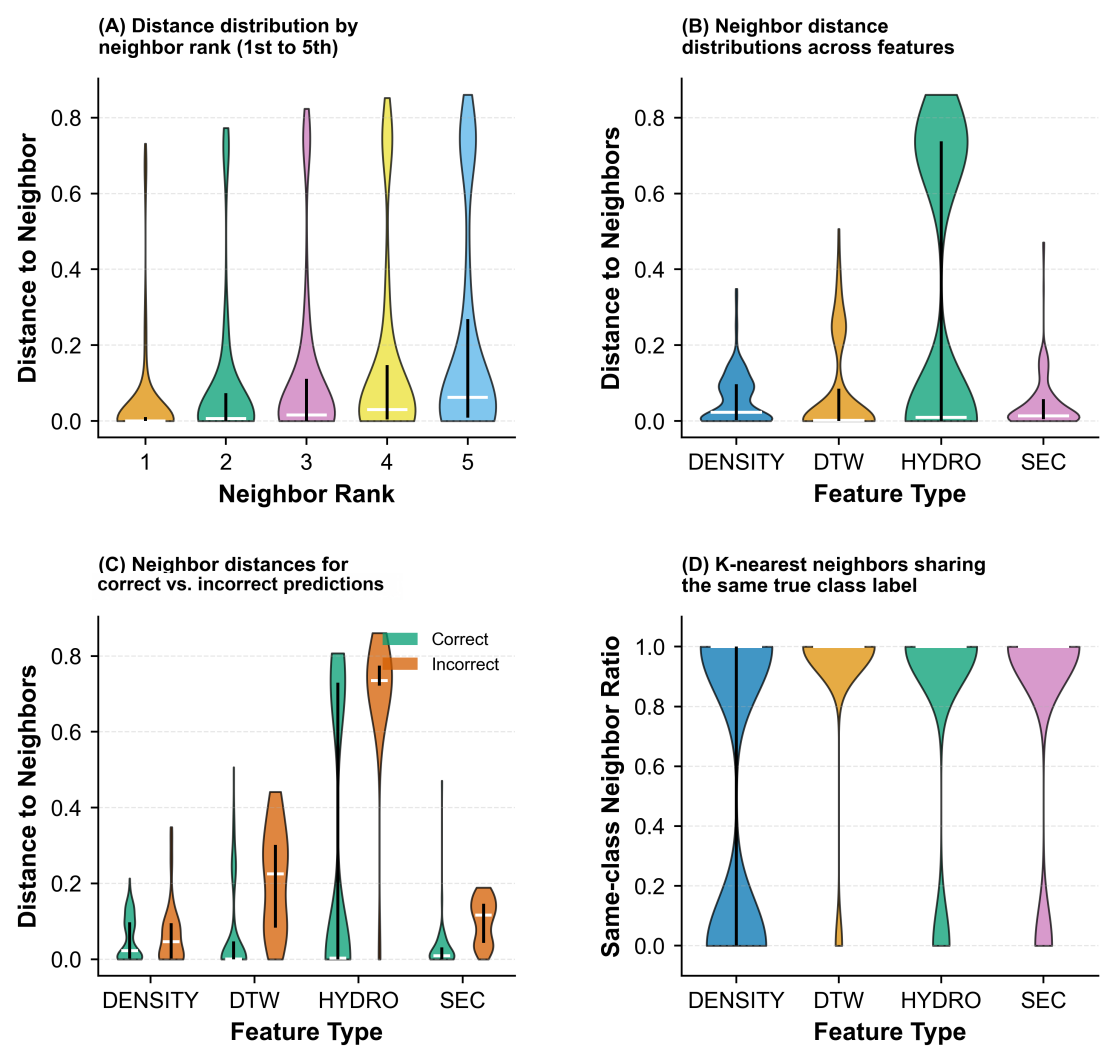
Neighbor-based classification analysis on the single-chain protein dataset. (**A**) Distance distribution by neighbor rank. (**B**) Feature-wise neighbor distances. (**C**) Prediction accuracy vs. distance: Correct (green) vs. incorrect (orange). (**D**) Same-class neighbor consistency across features (DENSITY, DTW, HYDRO, SEC).

Figure 10 examines the quality and characteristics of neighbor relationships in greater detail. Panel (A) demonstrates how distance to neighbors increases progressively from the first- to the fifth-nearest neighbor, with similar growth patterns across all features. Panel (B) highlights the substantial differences in distance scales among features, with HYDRO exhibiting significantly larger distances compared to other features. Panel (C) compares prediction accuracy against distance to neighbors, revealing that incorrect predictions (orange) often correspond to larger distances for DTW and HYDRO features, while DENSITY and SEC show more compact distance distributions regardless of prediction correctness. Panel (D) presents the same-class neighbor ratio, which quantifies the proportion of neighbors sharing the same class label. All features achieve high same-class neighbor ratios (predominantly above 0.8), suggesting that the K-nearest neighbor approach effectively captures local class structure in the feature space.

These comprehensive analyses collectively demonstrate that different features exhibit distinct distance characteristics and classification behaviors. The Majority Voting ensemble leverages these complementary properties to achieve superior performance by combining the strengths of individual base models.

As shown in Table 2, our distance-based ensemble (“Designed Features + Majority Voting”) delivers near state-of-the-art performance on the single-chain dataset under LOOCV, achieving 96.43% accuracy with a strong precision–recall balance (precision 97.73%, recall 92.86%, F1 94.99%). It surpasses all PseAAC-based methods and the G-GAP models paired with KNN and SVM, and attains the highest precision among methods with recall ≥90%. While we acknowledge that the G-GAP + Logistic Regression benchmark achieved the highest accuracy (98.21%), our approach offers a more favorable precision–recall profile. Importantly, this strong and interpretable performance is achieved with minimal training overhead, utilizing only a simple KNN framework and a biologically grounded feature space. To improve the readability, we bold two kinds of numbers in the table: those indicating the best performance and those corresponding to our own model.

Notice that the significant performance disparity observed for the PseAAC feature set—where KNN yielded only 78.57% accuracy compared to 94.64% for SVM/LR—is a direct consequence of the “curse of dimensionality.” Our use of 50-dimensional features on a small dataset of 56 samples creates a high-dimensional, sparse space where the distance-based neighborhood concept fundamental to KNN becomes unreliable. In contrast, the regularization inherent in SVM and Logistic Regression enables these models to identify a robust decision boundary by implicitly focusing on the most relevant features, thus explaining their superior performance. This conclusion is further corroborated by the results in Table 3, which show that ‘PseAAC + KNN’ performs better on the larger, whole Aspirin protein dataset than on the smaller single-chain subset, underscoring the impact of sample size in high-dimensional space.

#### 2.1.3. Performance on Whole Protein Dataset

After our “Designed Features + Majority Voting” approach achieved success on the single-chain protein dataset, we then applied it to a disassembled protein dataset. The results of our model’s performance are presented in detail.

**Figure 11:**
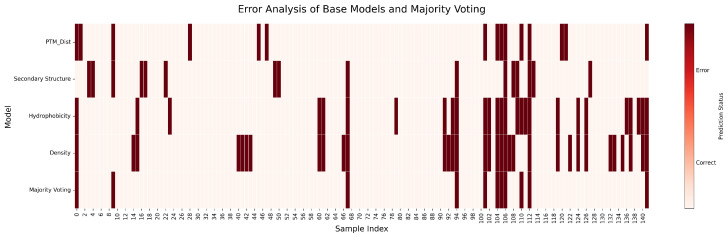
Error analysis of base models and Majority Voting on the whole Aspirin protein dataset. Each row represents a model, and each column corresponds to a test sample. Dark red indicates errors; light colors indicate correct predictions.

An extended error analysis across the entire dataset in Figure 11 confirms the complementary nature of the base learners. The heat map illustrates that while certain samples prove challenging for most models, the individual error profiles remain largely uncorrelated. This is particularly evident with the Density model, which again correctly identifies samples that are systematically missed by otherwise stronger predictors (PTM Dist, Secondary Structure). This observed diversity in predictive behavior is the cornerstone of successful ensembling. The Majority Voting model leverages this diversity to produce a more robust and accurate classification, as its own error profile is sparser than that of any individual constituent model.

The ablation study was conducted to demonstrate the necessity of the Density model. On this dataset, the result shows that with the Density model, the Majority Voting model performs at the accuracy of 92.25%, compared to an accuracy of 91.55% when the Density model is subtracted. This ablation study strongly supports the idea that it is of great necessity to keep the Density model in the ensemble model.

**Figure 12:**
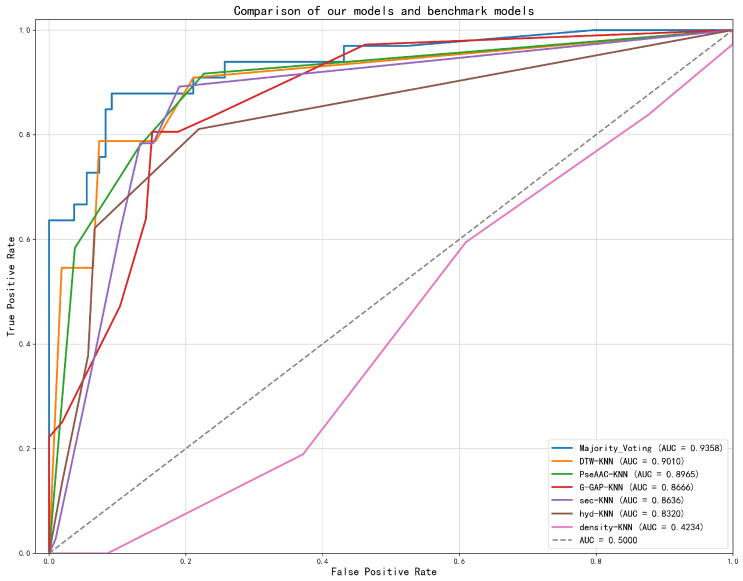
ROC curves comparing the performance of individual feature models (DTW-KNN, G-GAP-KNN, PseAAC-KNN, hyd-KNN, sec-KNN, density-KNN), the Majority Voting ensemble model, and a random baseline. The Majority Voting model achieves the highest AUC of 0.9358 on the whole Aspirin protein dataset.

Figure 12 demonstrates the ROC analysis of the proposed models and benchmarks. The Majority Voting ensemble achieved a superior AUC of 0.9358, markedly outperforming all constituent benchmark models, including PseAAC-KNN (AUC = 0.8965) and G-GAP-KNN (AUC = 0.8666).

**Figure 13:**
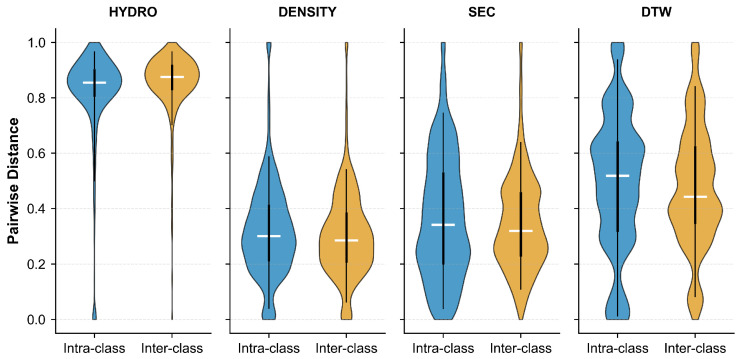
Violin plots illustrating pairwise distance distributions across different feature representations on the complete Aspirin protein dataset. Each panel contrasts within-class distances (blue) against between-class distances (yellow) for HYDRO, DENSITY, SEC, and DTW features. White horizontal bars denote median values.

**Figure 14:**
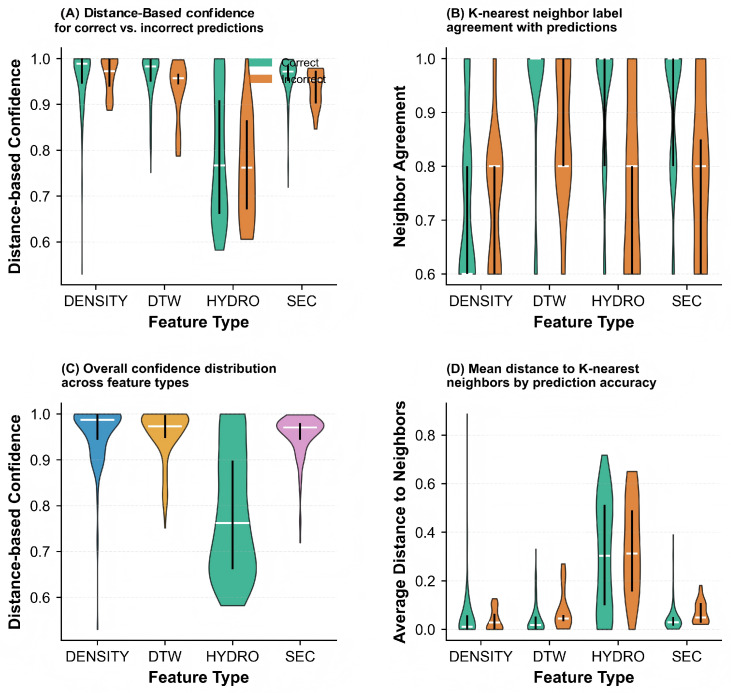
Comprehensive evaluation of prediction reliability and neighborhood characteristics across different features on the complete Aspirin protein dataset. (**A**) Confidence scores derived from distance metrics: Accurate predictions (green) versus erroneous predictions (orange). (**B**) Distribution of agreement levels among neighboring samples. (**C**) Comparative analysis of confidence scores across individual features. (**D**) Mean distances to nearest neighbors. Feature modalities include DENSITY, DTW, HYDRO, and SEC.

**Figure 15:**
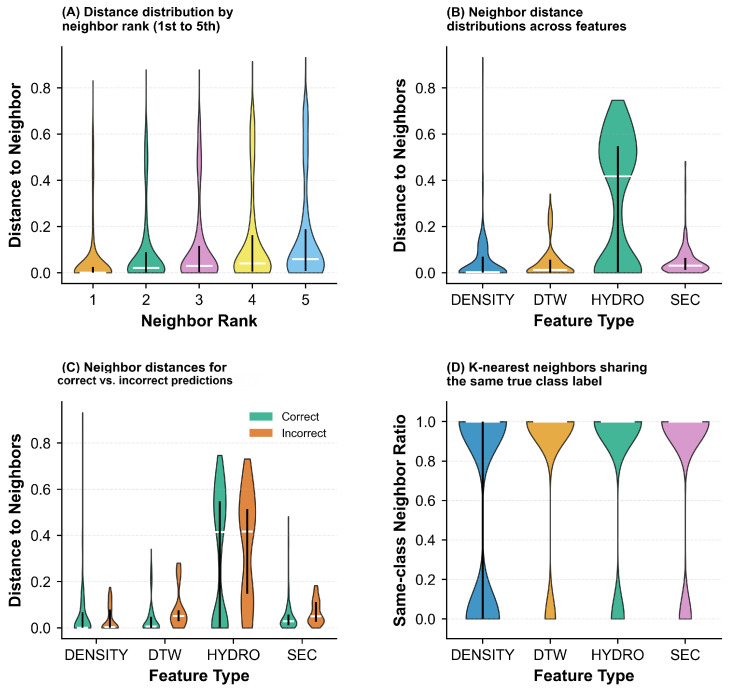
Detailed examination of neighborhood structure and quality in K-nearest neighbor classification on the complete Aspirin protein dataset. (**A**) Distance progression across neighbor rankings (1st to 5th nearest neighbors). (**B**) Comparison of distance magnitudes across different feature representations. (**C**) Relationship between prediction correctness and neighbor proximity: Accurate (green) versus inaccurate (orange) classifications. (**D**) Proportion of same-label neighbors across features (DENSITY, DTW, HYDRO, SEC).

To validate the generalizability of the observed patterns, we extended the analysis to the complete Aspirin protein dataset, which includes both single-chain and multi-chain proteins. The results are presented in Figure 13, Figure 14, and Figure 15.

Figure 13 illustrates the pairwise distance distributions across four feature representations: Hydrophobicity (HYDRO), Density (DENSITY), Secondary Structure (SEC), and DTW-based distance (DTW). Compared to the single-chain dataset, the complete dataset exhibits similar trends but with notable differences in separation quality. HYDRO maintains strong class separability with minimal overlap between within-class (blue) and between-class (yellow) distributions. DTW also demonstrates good discriminative ability, with inter-class distances predominantly exceeding intra-class distances. However, DENSITY and SEC show substantial overlap, suggesting that these features alone may have limited effectiveness in distinguishing between classes when multi-chain proteins are included. The increased complexity of multi-chain structures likely contributes to the larger variance observed in the distance distributions.

Figure 14 provides a comprehensive evaluation of prediction reliability across the complete dataset. Panel (A) presents the distance-based confidence scores, where accurate predictions (green) consistently achieve higher confidence values than erroneous predictions (orange) for all features, though the margin varies. Notably, HYDRO exhibits the most pronounced separation between correct and incorrect predictions, indicating its robustness in confidence estimation. Panel (B) displays the K-nearest neighbor label agreement distribution, revealing that accurate predictions generally correspond to higher neighbor agreement, particularly for HYDRO and DTW features. Panel (C) compares the overall confidence distributions across feature types, showing that DENSITY and DTW maintain relatively stable high-confidence regions, while HYDRO demonstrates broader variability, with a substantial proportion of samples exhibiting lower confidence scores. Panel (D) examines the mean distances to K-nearest neighbors, where correctly classified samples tend to have smaller average neighbor distances across all features, confirming that accurate predictions typically reside in more homogeneous regions of the feature space.

Figure 15 further investigates the neighborhood structure characteristics in the complete dataset. Panel (A) depicts the distance progression from the first- to the fifth-nearest neighbor, demonstrating a gradual increase in distance with neighbor rank across all features. The relatively smooth progression suggests well-structured local neighborhoods in the feature spaces. Panel (B) compares the magnitude of neighbor distances across features, revealing that HYDRO produces substantially larger distances compared to other features, which may reflect its different scaling properties or the higher dimensionality of its representation. Panel (C) analyzes the relationship between prediction correctness and neighbor proximity, showing that for HYDRO and DTW, incorrect predictions (orange) are often associated with larger neighbor distances, indicating that misclassified samples tend to be outliers or located in sparse regions. In contrast, DENSITY and SEC show more compact distance distributions regardless of prediction accuracy, suggesting that distance alone may not be a reliable indicator of prediction quality for these features. Panel (D) presents the proportion of same-label neighbors (same-class neighbor ratio), where all features achieve predominantly high ratios above 0.8, demonstrating that the K-nearest neighbor approach effectively captures local class structure even in the more complex complete dataset.

Comparing the results between the single-chain dataset (Figure 8, Figure 9, and Figure 10) and the complete dataset (Figure 13, Figure 14, and Figure 15), we observe that the fundamental patterns remain consistent, validating the robustness of our feature representations and ensemble approach. However, the inclusion of multi-chain proteins introduces additional complexity, resulting in increased variance in distance distributions and slightly reduced separation between classes for some features. Despite these challenges, the Majority Voting ensemble continues to leverage the complementary strengths of individual features, maintaining high classification performance across both datasets. This demonstrates the effectiveness and generalizability of our proposed approach for protein classification tasks.

Our analysis reveals a critical synergy within the feature ensemble. Individually, features such as DENSITY and DTW show limited discriminative power, evidenced by the substantial overlap in their intra-class and inter-class distance distributions across both datasets (Figure 8 and Figure 13). However, this belies their essential function in the integrated model. Our error analysis demonstrates that the DENSITY feature plays a vital complementary role, correcting systematic errors from other predictors within the Majority Voting ensemble. This illustrates a key principle: the ensemble’s success stems not from aggregating individually potent features, but from the synergistic compensation among features with unique error profiles. The value of a feature, therefore, is best measured by its marginal contribution to the collective, a strategy central to our model’s robust performance.

As shown in the Table 3, our model achieves an accuracy of 92.25%, which is more than 4 percentage points higher than the next-best models (PseAAC + SVM and G-GAP + SVM), both at 88.03%. In terms of the F1 Score, which provides a balanced measure of precision and recall, our model obtains a score of 88.23%. This result substantially outperforms all other methods, with the best-performing baseline (PseAAC + SVM) reaching only 76.06%.

Notably, our model also achieves the best balance and the highest values for both precision (92.27%) and recall (85.45%). This indicates that our framework is not only highly precise in its positive predictions but also effective at identifying the majority of true positive cases, demonstrating a comprehensively superior performance. These results clearly validate the effectiveness and robustness of our designed features and ensemble strategy for the task of protein druggability prediction.

## 3. Discussion

Our proposed model establishes a new state of the art in predicting protein druggability, unequivocally surpassing all conventional sequence-based benchmarks in our study. It achieved remarkable AUCs of 0.9634 and 0.9358 on the single-chain and multi-chain datasets, respectively, demonstrating a clear superiority over established methods like PseAAC and G-GAP. This breakthrough underscores the power of our novel multi-modal approach, which integrates structural and post-translational data to capture the intricate determinants of a protein’s druggable potential. At its core, this work introduces a more biologically coherent and actionable protein representation, paving the way for more efficient and accurate drug discovery.

Furthermore, the creation and public release of our curated dataset represents a significant contribution to the field. Existing public benchmarks for druggability prediction are scarce and often limited to sequence-level information. Our dataset will not only serve as a much-needed, high-quality standard for future methods’ development but also enable the broader community to explore novel structure–function relationships beyond the scope of this study. This work addresses a critical gap, fostering more reproducible and comparable research in computational pharmacology. We acknowledge, however, one primary limitation. The sample size of our dataset, while meticulously curated, remains modest due to the labor-intensive nature of manual data collection. This could potentially limit the statistical power of our findings and the generalizability of the model to unseen protein families.

To rigorously assess the statistical significance of our findings and address the potential influence of our modest sample size (*N* = 142) under leave-one-out cross-validation (LOOCV), we performed a comprehensive permutation test (1000 rounds). For each permutation, we randomly shuffled the class labels while keeping the feature matrices fixed, then re-executed the complete model pipeline to obtain the ensemble accuracy, thereby constructing a null distribution under the assumption of no true feature–label association. The empirical *p*-value was computed using the formula p=(b+1)/(m+1), where *b* represents the number of permutations yielding accuracy equal to or exceeding that of the true-label model, and m=1000 is the total number of permutations. This test challenged the null hypothesis that our model’s performance is attributable to chance. Our model, trained on true labels, achieved an accuracy of 92.25%. In stark contrast, models trained on randomly permuted labels yielded an average accuracy of only 76.80%, with the single best-performing random trial reaching a maximum accuracy of just 83.10%. The resulting *p*-value of observing our model’s performance by chance was p≤0.001. This allows us to confidently reject the null hypothesis, providing strong statistical evidence that our model learned genuine, non-random patterns from the data rather than overfitting to noise. While future work will focus on expanding the dataset to improve generalizability, this analysis robustly demonstrates that the results obtained from our current sample are statistically significant.

To fully unlock the potential of our framework, our immediate future work will focus on developing a robust, automated data acquisition and analysis pipeline. This will not only allow us to systematically expand the dataset’s size and scope with far greater efficiency but also enable a more thorough validation of our model across diverse protein families, especially those of high therapeutic interest. Looking forward, our ultimate goal is to translate this research into a tangible asset for the drug discovery community: a publicly accessible web server or downloadable software package. This tool will empower researchers to automatically featurize and predict the druggability of their own proteins of interest, streamlining the critical processes of target identification and validation. In conclusion, this study presents a powerful new computational tool and a valuable data resource that together lay the foundation for a more efficient, structurally informed, and data-driven approach to early-stage drug discovery.

## 4. Methods and Materials

We embedded proteins into four feature-induced non-Euclidean metric spaces, ${\left\{\left(M_{i},d_{i}\right)\right\}}_{i=1}^{4}$, each representing a distinct aspect of protein features. Protein similarity was then assessed by computing the distances $d_{i}\left(\cdot ,\cdot \right)$ between different proteins within these spaces. This metric-space encoding distills complex protein data into an analyzable form, captures key protein characteristics, and greatly facilitates the downstream drug prediction task.

Within non-Euclidean metric spaces, a variety of classifiers can be applied. Among these, *K*-nearest neighbors (KNN) stands out as the simplest and most straightforward option. To clearly demonstrate the effectiveness of our proposed feature representation, we primarily employ KNN for evaluation. Nevertheless, our metric-based features are also compatible with and beneficial to other classifiers designed for non-Euclidean settings.

### 4.1. Dataset

#### Aspirin Data Acquisition and Processing

We obtained our Aspirin-binding protein samples from a public dataset in ChemBL, an extensive open-access bioactivity resource (https://www.ebi.ac.uk/chembl, accessed on 15 March 2025) used to retrieve drug-binding protein results. Specifically, from the “Target Prediction” section, we derived the proteins predicted to be active with a confidence level of 90% (the highest attainable), which we categorized as druggable proteins. Conversely, we also identified proteins predicted to be inactive with the same degree of confidence, which we used as non-druggable proteins. Utilizing protein disassembling methods (which will be discussed in detail in Section 2.1.1) and strictly eliminating the same samples, our manually organized dataset included 142 Aspirin-binding protein chains as samples in total, within which there were 106 negative samples and 36 positive samples.

In our work, we have collected the amino acid sequences for these samples, forming a significant component of our raw dataset. However, key information regarding secondary structures, proportions, and PTM sites is not publicly accessible. Consequently, we have embarked on the substantial task of manually compiling and organizing the proteins’ secondary structure proportions and PTM sites.

To obtain secondary structure data, we applied SOPMA alongside the NPS@ platform, yielding 142 records with 568 secondary structural proportion entries across individual proteins. In parallel, we profiled eight types of PTM sites per protein, identifying 3579 specific positions across 142 proteins.

Through data collection and cleaning, we have constructed a novel and comprehensive protein dataset that, for the first time, integrates PTM site information, secondary structure proportions, and protein sequences. Our dataset can serve as a benchmark for further research in protein druggability analysis.

**Figure 16:**
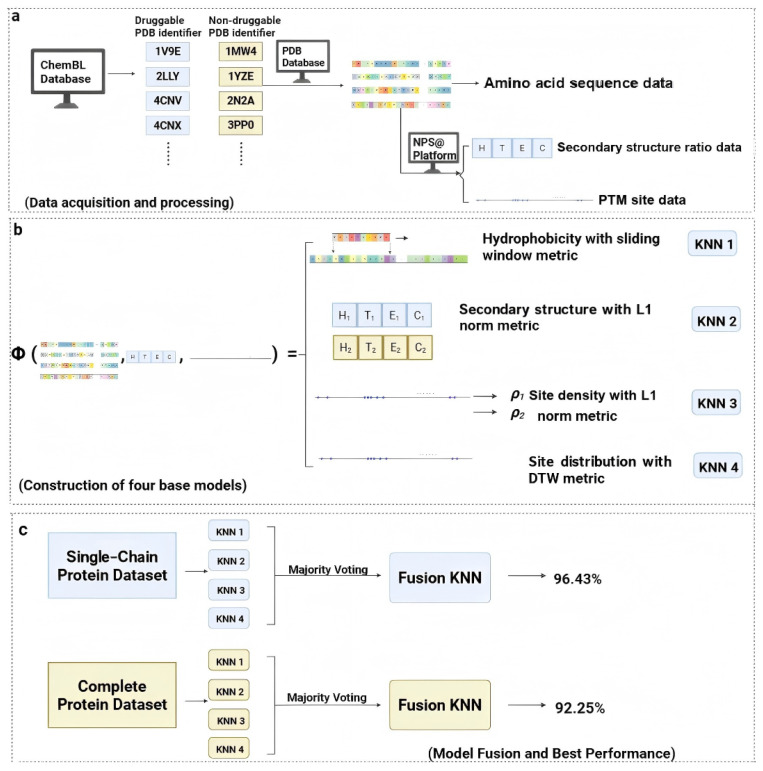
Workflow of our methods: (**a**) Data acquisition and processing; (**b**) feature extraction via metric space embedding Φ(·) and base model training; (**c**) model fusion.

To make full use of the protein samples derived from ChEMBL, we employed leave-one-out cross-validation (LOOCV) to evaluate model accuracy. In each iteration, one sample is held out as the test set, while the remaining samples are used for training (Figure 16).

### 4.2. Constructing Biologically Interpretable Features via Metric Embedding

In this section, we present four approaches for embedding proteins within non-Euclidean metric spaces. Subsequently, these spaces are integrated via an ensemble learning strategy.

#### 4.2.1. Metric Space $\left(M_{1},d_{1}\right)$: Hydrophobicity Feature via Sliding Window Metric

Grounded in biology, this feature prioritizes hydrophobicity due to its central role in protein self-assembly. The hydrophobic effect is a major driving force in protein folding, affecting the function of the proteins [20]. Structural analyses reveal that variations in the distribution of hydrophobic and hydrophilic regions directly influence the compactness as well as the secondary structure of proteins [21]. This interplay between hydrophobicity and electrostatics establishes a nuanced balance that profoundly influences protein folding and assembly. Thus, the selection of hydrophobicity as a feature is grounded in theoretical foundations and robust empirical evidence, significantly enhancing our understanding of protein functions and local structures.

There are several hydrophobicity scales in protein analysis, among which we choose the Kyte–Doolittle (KD) scale. The Kyte–Doolittle (KD) scale excels at detecting extended hydrophobic segments and transmembrane α-helices, enabling rapid, whole-sequence scanning. Compared with Hopp–Woods, which better identifies surface-exposed hydrophilic regions and putative epitopes, KD prioritizes hydrophobic segment detection. Relative to Wimley–White (thermodynamically grounded interfacial transfer energies) and the Eisenberg consensus scales (widely used for amphipathic helices), KD offers speed and generality but less interfacial specificity [22,23]. Versus octanol/water and GES scales, often used for membrane insertion and TM scoring, KD is more standardized and intuitively interpretable, making it a strong default baseline. It is therefore preferred for rapid, uniform profiling and preliminary localization of TM regions, hydrophobic cores, and candidate signal peptides, while specialized tools may be superior for detailed membrane energetics or signal peptide prediction.

To approximate structural similarity in relation to hydrophobicity—considering that the functionality of drug–protein bindings typically arises from specific regions, the specific position of which remains unknown—we chose to analyze the entire protein as our unit. This strategy effectively circumvents potentially ambiguous partitioning of the sequence. Then, we applied a sliding window approach in which the shorter chain $c_{i}$ traverses the longer reference chain $c_{j}$, which remains fixed as the alignment framework.

(1) $$D_{p,w}\left(c_{i},c_{j},t\right)={\left[\sum_{k=1}^{|c_{i}|}\frac{w_{k}}{W}{\left|H\left(c_{i}\left[k\right]\right)-H\left(c_{j}\left[st+k\right]\right)\right|}^{p}\right]}^{\frac{1}{p}},$$

where H(·) denotes the KD hydrophobicity value of residue *k*, $w_{k}$ is the weighting factor, *p* is the norm exponent, and *W* normalizes the total weight.

For computational efficiency, the window step size is set to two residues (i.e., s=2). At each window position, the hydrophobicity indices of aligned amino acids are compared; absolute differences are calculated for each residue pair and then summed to obtain a positional dissimilarity score. In our implementation, all positional weights were set to wk = 1 and the Lp norm was fixed to the $L_{1}$ case (p = 1), because for compositional features like secondary structure proportions, the L1 norm’s linear summation of absolute differences directly quantifies the total percentage of the structure, avoiding the disproportionate penalization of a single large deviation in one structural class that occurs with the squared-error-based $L_{2}$ norm, thus providing a more balanced and biologically interpretable measure of global fold dissimilarity. To facilitate comparison across different chain lengths and mitigate the influence of non-homologous regions, this value is normalized by dividing by the length of the shorter chain (i.e., $W=|c_{i}|$).

After the sliding step, the minimum sum across all window alignments is selected as the unnormalized similarity metric, as this captures the optimal alignment between the two hydrophobicity profiles, which is shown below:

(2) $$d_{\mathrm{hyd}}\left(c_{i},c_{j}\right)=\min_{t=0,\;\ldots ,\;\left\lfloor (|c_{j}|-|c_{i}|)/s\right\rfloor }D_{p,w}\left(c_{i},c_{j},t\right).$$

**Figure 17:**
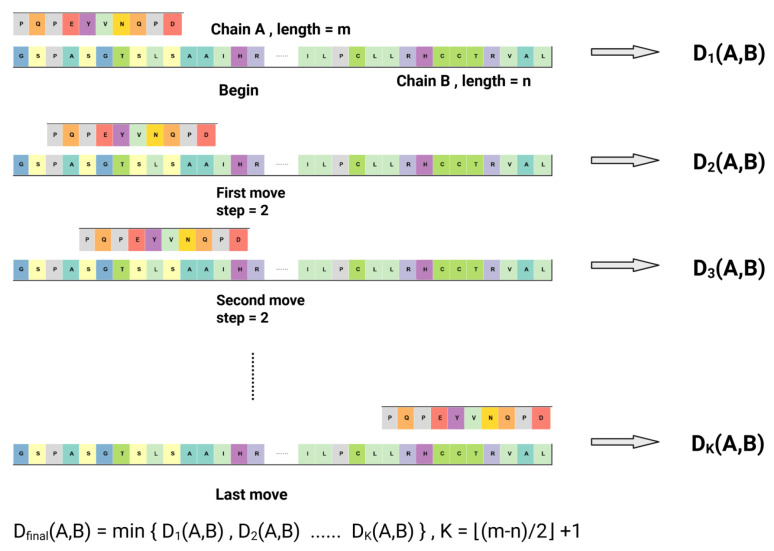
The principle of the hydrophobicity feature with a sliding window metric. This metric captures the most similar local structure part from a hydrophobicity perspective.

The whole process is illustrated in Figure 17.

#### 4.2.2. Metric Space $\left(M_{2},d_{2}\right)$: PTM Density and $l_{1}$ Norm Metric

Biologically motivated and justified, this feature exploits the fact that the linear density of modification sites along a polypeptide sequence reflects its three-dimensional structure. The differential phosphorylation density between ordered and disordered segments significantly correlates with domain stability and architecture [24]. Systems-level analyses further reinforce this principle, showing that clusters of high phosphorylation density—rather than isolated sites—are statistically co-localized with specific structural motifs and functional hubs [25,26]. Thus, the linear density of phosphorylation sites emerges not as a contingent biological feature but as a high-fidelity descriptor of tertiary and quaternary structure.

Given that the exact drug–target binding region is unknown to us, we consider the global site abundance of the chosen PTM type density, thereby avoiding unreasonable partitioning as well as the loss of interpretability. For each protein, the relative abundance of each PTM type is quantified by normalizing the site count to the effective length of the sequence, where the latter is rigorously defined as the residue position of the most terminal PTM, irrespective of modification class. This PTM-centric delineation of sequence boundaries is motivated by the frequent colocalization of PTMs with functional drug-binding regions, permitting the exclusion of terminal segments devoid of modification sites.

**Figure 18:**
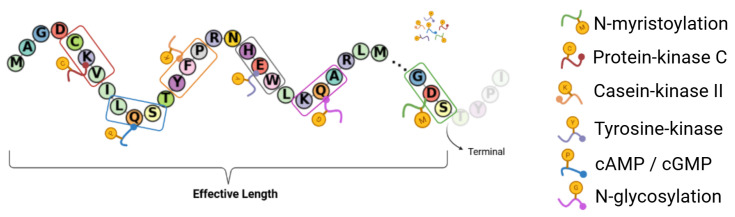
Post-translational modification (PTM) sites and effective length. The diagram shows a protein sequence where specific residues undergo PTMs. These modifications are decoded by the legend on the right and include N-myristoylation (M), protein-kinase C phosphorylation (C), casein-kinase II phosphorylation (K), tyrosine-kinase phosphorylation (Y), cAMP/cGMP-dependent protein-kinase phosphorylation (P), and N-glycosylation (G). The ‘effective length’ is defined as the sequence span between the first and last detected PTM site. Residues outside this range (Terminal, faded) are excluded from the analysis, focusing on the functionally relevant region.

In addition to the six types of common PTMs illustrated in Figure 18, we specifically focus on the Ubiquitin-Specific Protease (USP) domain. The basic mechanism for this focus is that, as a member of the USP family, CYLD plays a crucial role in regulating various biological processes, including inflammatory responses [27]. This regulation is particularly relevant to the effects of Aspirin.

Subsequently, we assess the pairwise divergence in PTM landscapes by computing the $L_{1}$ norm on the combined seven-dimensional vector, which comprises the six PTM density features and the USP density, yielding a distance that directly reflects the overall disparity in modification patterning between two proteins. The $L_{1}$ norm offers a robust and interpretable measure of aggregate absolute differences, making it especially well-suited for capturing direct, meaningful divergences in PTM density profiles. The employment of site densities, rather than raw counts, inherently normalizes this measure, enabling robust comparison across proteins of disparate effective lengths.

(3) $$D_{\mathrm{PTM}}^{\left(p\right)}\left(x,y\right)={\left(\sum_{k=1}^{n}{\left|\rho_{x}^{\left(k\right)}-\rho_{y}^{\left(k\right)}\right|}^{p}\right)}^{1/p},$$

where $\rho_{x}^{\left(k\right)}$ and $\rho_{y}^{\left(k\right)}$ denote the density of the *k*-th PTM type for protein chains x and y, respectively; *n* is the total number of PTM types considered (here, n=7); and *p* is the order of the norm (with p=1 corresponding to the $L_{1}$ norm used in this study).

#### 4.2.3. Metric Space $\left(M_{3},d_{3}\right)$: PTM Distribution via Dynamic Time Warping (DTW) Metric

Developed on biologically substantiated principles, this feature leverages the close link between the spatial distribution of PTM sites along a polypeptide and the protein’s local 3D architecture to inform structure and function. Phosphoproteomic analyses show that phosphorylation sites are strategically located within or near specific structural domains, particularly in solvent-accessible regions, emphasizing their role in localized protein function [24]. This relationship is pronounced in structurally plastic regions, such as intrinsically disordered regions (IDRs), where dense phosphorylation patterns reveal local conformational transitions that influence protein folding and interaction interfaces [28]. Additionally, systems-level analyses indicate that clusters of modification sites define distinct functional modules and align with specific local folding patterns, effectively forming a “PTM code” at the local structural level [25]. These regions often coincide with critical structural interfaces vital for protein stability and biomolecular interactions [26]. Consequently, the arrangement of these modification sites serves as a direct descriptor of local protein functionality and regulatory potential.

To rigorously compare PTM spatial distribution between protein chains, we first map each PTM site to a relative position, defined as the absolute residue index divided by the effective sequence length (determined by the most terminal PTM site). Owing to the nature of PTMs, which frequently span short oligopeptide segments (3–8 residues), we standardize the assignment by defining the central residue of each modified interval as its representative position. Specifically, for a modified peptide region spanning residue indices $\left[a_{i},b_{i}\right]$, its center is

(4) $$c_{i}=\left\lfloor \frac{a_{i}+b_{i}}{2}\right\rfloor ,$$

where $c_{i}$ denotes the index of the central residue. The effective sequence length $L_{\mathrm{eff}}$ is determined as

(5) $$L_{\mathrm{eff}}=\max\left\{b_{1},b_{2},\ldots ,b_{n}\right\}$$

and each PTM’s relative position is calculated as

(6) $$r_{i}=\frac{c_{i}}{L_{\mathrm{eff}}}.$$

For each modification type, these normalized PTM positions form a one-dimensional distribution profile. To assess similarity between proteins, we apply the DTW algorithm to seek an optimal nonlinear alignment between two sequences by minimizing the cumulative distance along a flexible warping path.

Specifically, for protein A and protein B with relative PTM position sequences $R^{\left(A\right)}=\left(r_{1}^{\left(A\right)},\ldots ,r_{m}^{\left(A\right)}\right)$ and $R^{\left(B\right)}=\left(r_{1}^{\left(B\right)},\ldots ,r_{n}^{\left(B\right)}\right)$, the DTW cost matrix *D* is recursively defined as

(7) $$D_{i,j}=d\left(r_{i}^{\left(A\right)},r_{j}^{\left(B\right)}\right)+\min\left(D_{i-1,j},D_{i,j-1},D_{i-1,j-1}\right),$$

with *d* being a pointwise distance, typically d(r,s)=|r−s|, and $D_{0,0}=0$ while $D_{i,0},D_{0,j}=\infty$ for i>0 or j>0. The DTW alignment distance is the total minimum cost.

(8) $$\mathrm{DTW}\left(R^{\left(A\right)},R^{\left(B\right)}\right)=D_{m,n}.$$

For PTM types missing from one or both proteins, we assigned a pseudo-position value of 2, exceeding the normalized [0, 1] range, as a penalty. In such a case, for any comparison involving a non-existent PTM, the DTW distance degenerates into pointwise distance, which is

(9) d(r,2)=|r−2|,r∈[0, 1]

This penalization reflects the increased likelihood of underlying structural divergence when a PTM is absent, while its magnitude is calibrated to avoid disproportionate influence from any single PTM type.

The DTW alignment cost was calculated independently for each of the six major PTM categories plus USP domain. Summing these costs across all modification types ($l_{1}$ norm) yields the final metric, providing a comprehensive and sensitive quantification of PTM spatial divergence between protein chains:

(10) $$D_{\mathrm{final}}=\sum_{k=1}^{n}D_{k},$$

where $D_{k}$ is the DTW cost for the *k*-th PTM type.

DTW effectively accommodates local insertions, deletions, and non-uniform positional shifts, enabling robust comparison of PTM patterns despite variability in protein length or PTM site distribution.

#### 4.2.4. Metric Space $\left(M_{4},d_{4}\right)$: Secondary Structure Proportion via $l_{1}$ Norm Metric

**Figure 19:**
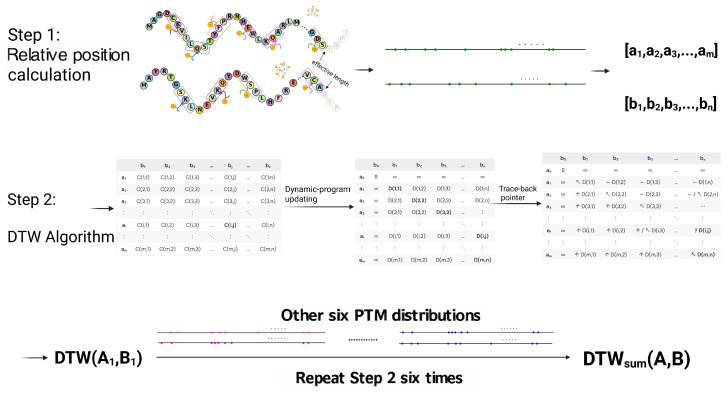
Workflow for calculating PTM similarity using Dynamic Time Warping (DTW). **Step 1:** For each of the seven PTM types, the relative positions of its occurrences within the ‘effective length’ of two proteins (A and B) are extracted into ordered sequences (e.g., $\left[a_{1}\ldots a_{m}\right]$ and $\left[b_{1}\ldots b_{n}\right]$). **Step 2:** For each PTM type, DTW finds the optimal alignment path between these position sequences, yielding a minimum cumulative distance, or alignment cost (DTW($A_{i},B_{i}$)). This step is repeated for all six PTMs plus one domain, USP. The individual costs are then summed into a final distance score, DTW_sum(A,B), which quantifies the overall dissimilarity of the PTM patterns between the two proteins.

The biological background for designing this feature as well as utilizing the $l_{1}$ norm is given as follows. The proportional makeup of secondary structure elements—α-helices, β-sheets, β-turns, and random coils—encodes essential information about protein fold, stability, and function. The relative abundance of these motifs is predictive enough to inform fold classifications like CATH [29] and correlate with chain compactness in molecular dynamics simulations [30]. Contemporary structure-prediction pipelines, such as DeepCNF and AlphaFold, leverage secondary structure probabilities as critical intermediates for reconstructing atomic coordinates from sequences [7,31]. Therefore, the percentages of helices, sheets, and loops are not merely descriptive; they reflect protein folding pathway, local stability, and biological activity, making them indispensable quantitative descriptors (Figure 19).

The rigorous technique for evaluating the distance between two protein chains is given as follows: For each disassembled protein chain *c*, its predicted secondary structure profile is represented as a four-dimensional vector:

$$f\left(c\right)=[f_{H}\left(c\right),\;f_{E}\left(c\right),\;f_{T}\left(c\right),\;f_{C}\left(c\right)],$$

where $f_{H}\left(c\right)$, $f_{E}\left(c\right)$, $f_{T}\left(c\right)$, and $f_{C}\left(c\right)$ denote the respective fractions of alpha-helix (H), beta-strand (E), turn (T), and coil (C) components in chain *c*, with

$$f_{H}\left(c\right)+f_{E}\left(c\right)+f_{T}\left(c\right)+f_{C}\left(c\right)=1.$$

To quantitatively assess the similarity between two proteins $c_{i}$ and $c_{j}$, we define the distance between their secondary structure profiles as the $l_{1}$ norm between their feature vectors:

$$d_{\mathrm{ss}}\left(c_{i},c_{j}\right)=\sum_{s\in \{H,E,T,C\}}\left|f_{s}\left(c_{i}\right)-f_{s}\left(c_{j}\right)\right|\;.$$

This metric directly reflects the absolute compositional divergence of secondary structure between proteins, enabling effective structural similarity comparison in the absence of high-resolution experimental data.

#### 4.2.5. Fusion of Metric Space by KNN Model Ensemble

##### Majority Voting

In the Majority Voting strategy for this binary classification task, consider the four classifiers $C_{1},C_{2},C_{3},\;\mathrm{and}\;C_{4}$. Each classifier predicts a class label $C_{i}\in \left\{0,1\right\}$. The final predicted class is determined by the label receiving the most votes:

(11) $$\hat{y}=\underset{c\in \{0,1\}}{arg max}\sum_{i=1}^{4}I\left(C_{i}=c\right),$$

where I(·) is the indicator function. Majority voting is particularly effective when models perform similarly, enhancing the reliability of decisions.

## 5. Conclusions

Our work introduces four effective strategies for protein representation in non-Euclidean metric space, culminating in a computationally efficient KNN classifier for druggability assessment—a crucial first step in modern drug discovery. To establish a robust foundation, we first validated our model on a benchmark dataset of single-chain proteins under leave-one-out cross-validation (LOOCV), where it demonstrated strong performance amidst minimal structural ambiguity and competitive results against strong feature engineering baselines. We then successfully extended this approach to classify the druggability of complex multi-chain proteins using a data disassembling technique. This achieved consistently strong results—and superior performance relative to widely used sequence-only baselines in the whole-protein setting—confirming the model’s generalizability across diverse structural contexts relevant to therapeutic development.

Remarkably, in the whole-protein setting, our framework achieves superior predictive accuracy relative to sequence-only baselines while utilizing a substantially designed feature set. This efficiency underscores the potent informational content of our selected descriptors. These features not only possess strong interpretive value for identifying and prioritizing druggable targets but also serve as reliable indicators for protein functional annotation, highlighting their dual utility in both drug discovery and fundamental biological research.

However, we acknowledge a key limitation impacting immediate widespread use: the current approach relies on curated experimental data for secondary structure proportions and PTMs, which may not always be accessible. To address this, we plan to connect our pipeline to advanced prediction services. However, we recognize that using predicted features introduces challenges: predictions are inherently less accurate, they may be biased due to the training set composition of the prediction tools, and errors may propagate in our model. Future work will focus on systematically benchmarking the impact of feature prediction reliability, filtering low-confidence results, and integrating consensus from multiple tools in order to minimize error propagation and bias and thereby maintain the robustness and interpretability of our method. In this context, this initiative will create a complete end-to-end protein analysis pipeline, enhancing the model’s utility as a scalable tool for the pharmaceutical sciences. Further research will also focus on expanding the sample size to continue validating the power of our feature design across a wider range of drug target classes. Finally, we are committed to improving reproducibility by releasing versioned data sources and code, ensuring our method can serve as a reliable and transparent benchmark for future computational pharmacology studies.

## Figures and Tables

**Table 1:** Ablation study of the labeling strategy on model performance (LOOCV).

Labeling Strategy	Accuracy (%)	Precision (%)	Recall (%)	F1 Score (%)	ΔAcc. (%)
**Full Model (K-Means + Correcting Algorithm)**	**92.25**	**92.27**	**85.45**	**88.23**	–
Ablated Model (Raw K-Means Only)	89.44	89.23	81.92	84.70	−2.81

**Table 2:** Benchmark comparison on single-chain Aspirin protein dataset (LOOCV).

Method	Accuracy (%)	Precision (%)	Recall (%)	F1 Score (%)
**Designed Features + Majority Voting**	**96.43**	**97.73**	**92.86**	**94.99**
PseAAC + KNN	78.57	57.69	93.75	71.43
PseAAC + Logistic Regression	94.64	88.24	93.75	90.91
PseAAC + SVM	94.64	93.33	87.50	90.32
G-GAP + KNN	89.29	77.78	87.50	82.35
G-GAP + SVM	94.64	**100.00**	81.25	89.66
G-GAP + Logistic Regression	**98.21**	94.12	**100.00**	**96.97**

**Table 3:** Benchmark Comparison on disassembled Aspirin-binding protein dataset (LOOCV).

Method	Accuracy (%)	Precision (%)	Recall (%)	F1 Score (%)
**Designed Features + Majority Voting**	**92.25**	**92.27**	**85.45**	**88.23**
PseAAC + KNN	84.51	67.50	75.00	71.05
PseAAC + Logistic Regression	82.39	62.79	75.00	68.35
PseAAC + SVM	88.03	77.14	75.00	76.06
G-GAP + KNN	83.80	64.44	80.56	71.60
G-GAP + SVM	88.03	80.65	69.44	74.63
G-GAP + Logistic Regression	87.32	78.12	69.44	73.53

## Data Availability

Code and Data Availability: The constructed dataset and codes are publicly available through https://github.com/David-SUSTech/Non-Euclidean-protein-representation-for-druggability-prediction, accesed on 24 September 2025.

## Abbreviations

The following abbreviations are used in this manuscript:

PTM	Post-Translational Modification
DL	Deep Learning
ML	Machine Learning
CNN	Convolutional Neural Network
AAC	Amino Acid Composition
PseAAC	Pseudo-Amino-Acid Composition
NLP	Natural Language Processing
CTD	Composition–Transition–Distribution
HMM	Hidden Markov Model
SO	Sequence Order
PP	Physicochemical Property
KNN	K-Nearest Neighbors
KD	Kyte–Doolittle
USP	Ubiquitin-Specific Protease
DTW	Dynamic Time Warping
IDRs	Intrinsically Disordered Regions
RF	Random Forest
SVM	Support Vector Machine
ROC	Receiver Operating Characteristic Curve
AUC	Area Under the Curve
